# Nrf2 promotes NLRP3 inflammasome assembly and activation by Klf9-TXNIP axis

**DOI:** 10.1038/s41420-026-03219-3

**Published:** 2026-06-26

**Authors:** You Zhou, Hui You, Xin-Yu Xia, Ke Zhang, Hui Jiang, Liang-Hua Liu, Hong-Ping Liu, Ming-Cai Zhao, Su-Yu He

**Affiliations:** 1 Department of Medical Laboratory, Suining Central Hospital, Suining, China; 2 The Key Laboratory of Molecular Biology of Infectious Diseases Designated by the Chinese Ministry of Education, Chongqing Medical University, Chongqing, China; 3 Department of Ophthalmology, Suining Central Hospital, Suining, China; 4 The Biology Bank of Suining Central Hospital, Suining Central Hospital, Suining, China; 5 Digestive Disease Center, Suining Central Hospital, Suining, China

**Keywords:** Inflammasome, NOD-like receptors

## Abstract

NLRP3 inflammasome activation shows a crucial role in the innate immune response that triggers inflammation. Activation is controlled by two sequential steps: a priming step, followed by an assembly step. Nrf2 has been shown to promote NLRP3 inflammasome activation; however, the underlying mechanisms remain elusive. This study identifies Nrf2 as an encourager of NLRP3 inflammasome activation. We show that Nrf2 inhibition suppresses NLRP3 inflammasome activation in macrophages, including THP-1 cells and BMDMs. In addition, Nrf2 facilitates NLRP3 inflammasome assembly. Mechanically, Nrf2 increases Klf9 expression by binding to the Klf9 promoter. Klf9 stabilizes TXNIP by deubiquitination, thereby enhancing the TXNIP-NLRP3 interaction, which is required for NLRP3 inflammasome assembly and full activation. Finally, Nrf2 deficiency exerts a protective effect and alleviates NLRP3 inflammasome activation in mouse models of DSS-induced colitis and MSU crystals-induced acute gouty arthritis. Our study reveals a novel regulatory mechanism of NLRP3 inflammasome by Nrf2 and indicates Nrf2 may be a promising target for treating NLRP3 inflammasome-driven diseases.

## Introduction

NLRP3 (NLR family pyrin domain containing 3) inflammasome is a multi-protein cytosolic immune sensor, its components comprising NLRP3, PYCARD/ASC and caspase-1, which is activated upon extensive stimuli including pathogens, crystals, and exogenous and endogenous damage signals [1–5]. Upon activation, these components form a complex to induce activation of caspase-1 and subsequently contribute to the release of inflammatory cytokines and cell death. It has been proven that multiple diseases of humans, including obesity [6]. diabetes [7]. atherosclerosis [5]. Gout [8] and sepsis [9] are closely related to deviant NLRP3 inflammasome activation. Thus, further clarification of the mechanisms underlying NLRP3 inflammasome activation may help develop therapeutic strategies to treat these diseases.

NLRP3 inflammasome activation is a sequential process comprising two steps: a priming signal and an assembly signal. The priming signal is mediated by danger/damage-associated molecular patterns (DAMPs) or pathogen-associated molecular patterns (PAMPs), and facilitates the expression of components of the NLRP3 inflammasome. The assembly signal is elicited by strong stimuli, such as nigericin, ATP, amyloid-β, and crystals, and promotes the NLRP3 inflammasome assembly and full activation, which leads to IL-18 and IL-1β production [10]. Upon stimulation, various upstream cellular signals have been authenticated to be responsible for the assembly signal, such as potassium efflux [11]. chloride ion efflux [12]. lysosomal leakage [13]. mitochondrial damage, and excessive production of reactive oxygen species(ROS) [14]. The assembly process is a common pathway for NLRP3 inflammasome activation [11–14]. Thus, an accumulating body of research on the NLRP3 inflammasome assembly has been devoted to exploring these common pathways to uncover the cognate target in response to disparate stimuli [11–16].

Nuclear factor E2-related factor 2 (Nrf2)-mediated cellular antioxidant effects are critical for maintaining homeostasis by inducing antioxidant enzyme expression [17]. Nrf2 performs a protective role in multiple inflammatory diseases via its antioxidant function, including liver injury [18]. and sickle cell disease [19]. However, the role of Nrf2 in NLRP3 inflammasome activation remains debated. Even though oxidative stress is a key signaling conductor that promotes NLRP3 inflammasome activation, and Nrf2 is recognized as an antioxidant regulator, recent research has reported that Nrf2 is indispensable for NLRP3 inflammasome activation [20–22]. Silencing or deleting Nrf2 inhibits, rather than promotes, the NLRP3 inflammasome activation triggered by cholesterol crystals [20]. MSU [21]. ATP, or nigericin [22]. We previously reported that the suppressive effect of lipoxin A4 on MSU-induced NLRP3 inflammasome activation is determined by repressing Nrf2 [23]. However, how Nrf2 regulates NLRP3 inflammasome activation, especially the biological mechanisms, remains unclear.

In this study, we examined the impact of Nrf2 on NLRP3 inflammasome activation and explored the regulatory mechanism underlying targeting the Krüppel-Like Factor 9(Klf9)-Thioredoxin-interacting protein(TXNIP) axis. We describe an interesting appearance that Nrf2 promotes NLRP3 inflammasome activation by facilitating its assembly. Mechanistically, Nrf2 upregulates Klf9 expression by binding to the Klf9 promoter to increase its transcription. Klf9 interacts with TXNIP and stabilizes it by deubiquitinating it, thereby enhancing the TXNIP-NLRP3 interaction, which promotes NLRP3 inflammasome assembly and activation. Our study elucidates the specific mechanism by which Nrf2 promotes NLRP3 inflammasome activation, and Nrf2 may be a promising target for treating related diseases.

## Results

### Nrf2 promotes NLRP3 inflammasome activation

**Fig. 1:**
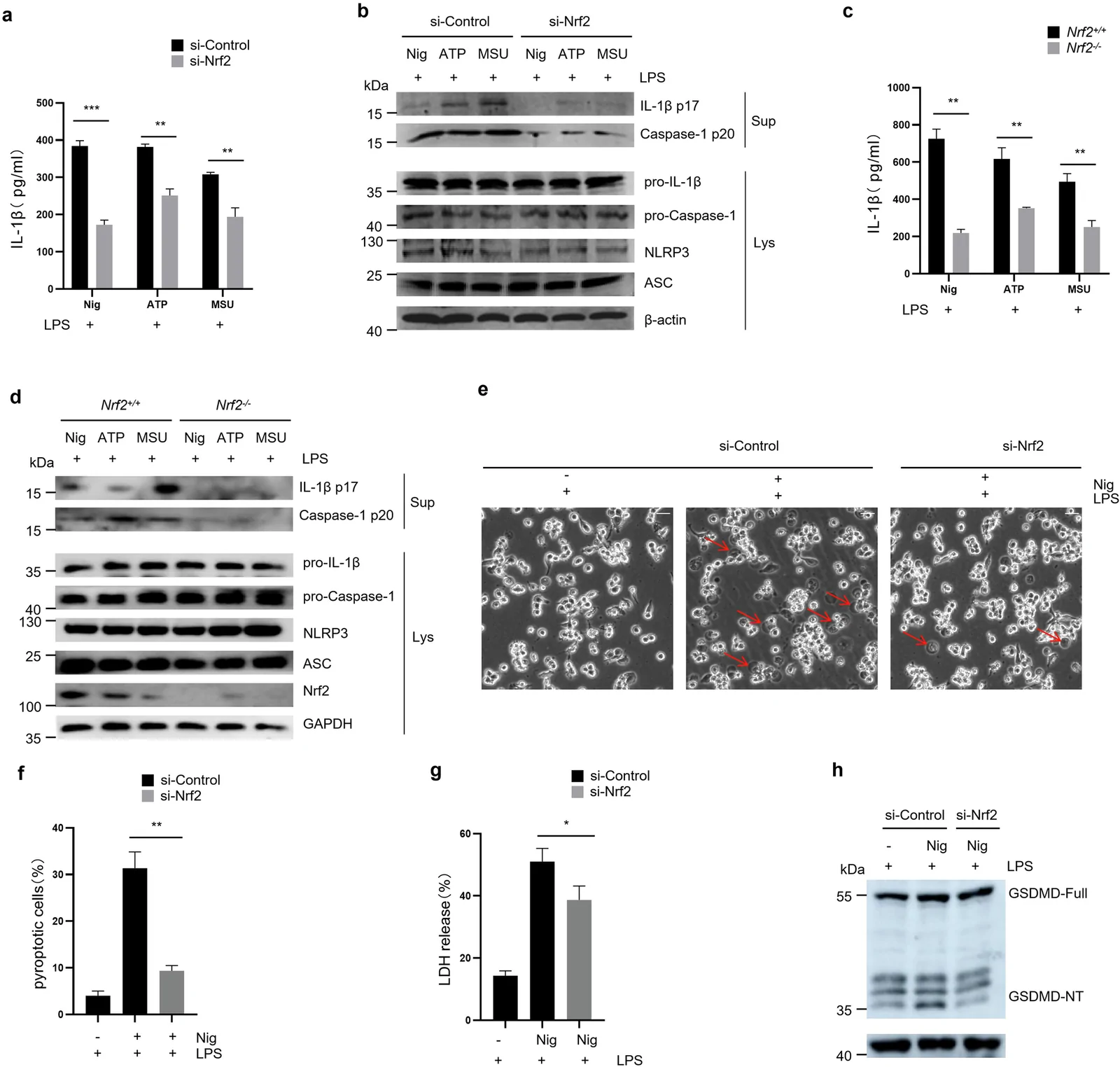
Nrf2 promotes NLRP3 inflammasome activation. **a** ELISA of IL-1β in supernatants from THP-1 cells silenced of Nrf2, primed with LPS, and followed by stimulation with Nigericin, ATP and MSU. **b** Immunoblot analysis of supernatants (Sup) or cell lysates(Lys) from THP-1 cells silenced of Nrf2, primed with LPS, and followed by stimulation with Nigericin, ATP and MSU. **c** ELISA of IL-1β in supernatants of BMDMs from Nrf2^+/+^ or Nrf2^–/–^ mice, primed with LPS, and followed by stimulation with Nigericin, ATP and MSU. **d** Immunoblot analysis of supernatants or cell lysates of BMDMs from Nrf2^+/+^ or Nrf2^–/–^ mice, primed with LPS, and followed by stimulation with Nigericin, ATP and MSU. **e**, **f** Image and counting of pyroptosis cells in THP-1cells silenced of Nrf2, primed with LPS, and followed by stimulation with Nigericin. Scale bar, 50μm. **g** LDH release of THP-1cells silenced of Nrf2, primed with LPS, and followed by stimulation with Nigericin. **h** Immunoblot analysis of GSDMD from THP-1cells silenced of Nrf2, primed with LPS, and followed by stimulation with Nigericin. Data are from three independent experiments (**a, c, f, g** Values are mean ± SD) or are representative of three independent experiments with similar results. Student' s *t*-test (**a, c, f, g**), *P <0.05, **P <0.01, ***P <0.001.

Previously, studies have reported that Nrf2 is required for NLRP3 inflammasome activation [20–22]. We evaluated the function of Nrf2 in the present study. To explore the physiological role of Nrf2 during NLRP3 inflammasome activation, we designed a small interfering RNA (siRNA) targeting human Nrf2 and transfected it into THP-1 cells (Supplementary Fig. 1a). In LPS-primed human THP-1 cells treated with nigericin, ATP, or MSU, IL-1β secretion was significantly decreased in Nrf2-silenced cells compared to that in control siRNA-transfected cells (Fig. 1a). Immunoblotting showed that silencing Nrf2 by siRNA suppressed IL-1β p17 in supernatants of THP-1 cells induced by LPS plus nigericin, ATP, or MSU (Fig. 1b). The positive effect of Nrf2 in regulating NLRP3 inflammasome activation was verified by using a macrophage model from an Nrf2-knockout mouse. BMDMs were primed with LPS and treated with nigericin, ATP, or MSU to activate the NLRP3 inflammasome. The secretion of IL-1β was depressed in Nrf2^–/–^ BMDMs compared to that in Nrf2^+/+^ BMDMs (Fig. 1c). Immunoblotting showed that IL-1β p17 in the supernatants of Nrf2^–/–^ BMDMs was also decreased (Fig. 1d). Given that the cleavage of caspase-1 is a decisive step in IL-1β maturation, we also explored the effect of Nrf2 on caspase-1 cleavage. Nrf2 silencing or deletion significantly attenuated caspase-1 cleavage in LPS-primed THP-1 cells and BMDMs treated with nigericin, ATP, or MSU (Fig. 1b, d). These results indicate that Nrf2 depression could suppress NLRP3 inflammasome-driven caspase-1 activation and IL-1β maturation.

In addition to the caspase-1-dependent IL-1β release, NLRP3 inflammasome activation can also contribute to GSDMD-mediated pyroptosis [24]. Some characteristic morphological changes related to pyroptosis in Nrf2-silenced THP-1 cells following LPS and nigericin treatment were decreased, including cell swelling and vacuoles around cells [25]. and the number of pyroptotic cells decreased (Fig. 1e, f). Next, an LDH release assay that was used to quantify pyroptosis showed that silencing Nrf2 suppressed the LDH release of THP-1 cells in response to nigericin (Fig. 1g). Consistent with the above results, knocking down Nrf2 blocked the formation of GSDMD-N (Fig. 1h), which induces the release of proinflammatory factors by promoting membrane rupture in cells. Collectively, these results demonstrate that Nrf2 promotes NLRP3 inflammasome activation.

### Nrf2 is required for NLRP3 inflammasome assembly

**Fig. 2:**
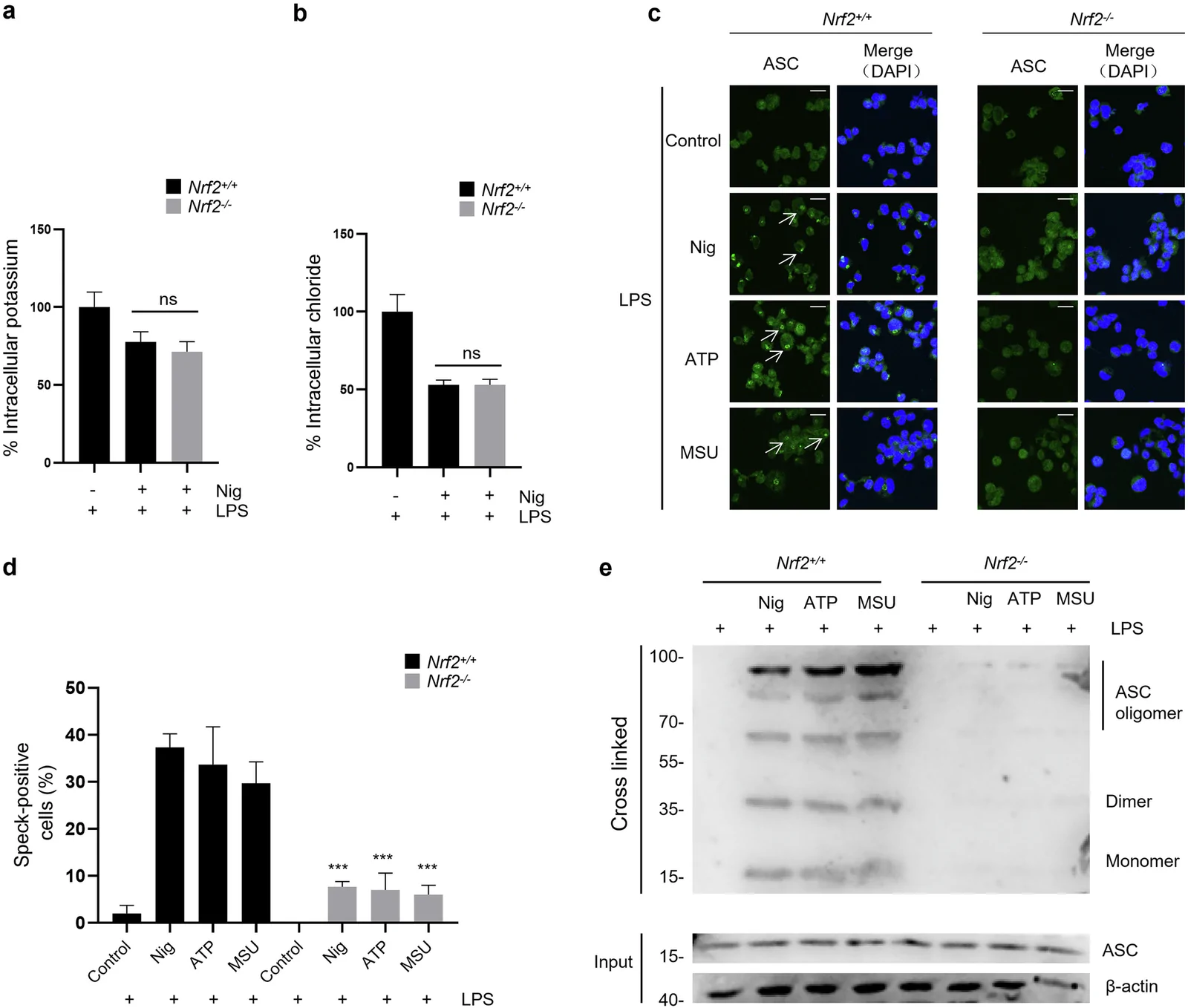
Nrf2 is required for NLRP3 inflammasome assembly. Qualification of the decrease of intracellular potassium (**a**) and chloride ion (**b**) from BMDMs from Nrf2^–/–^ mice, primed with LPS, and followed by stimulation with Nigericin. **c**, **d** Immunofluorescence analysis of ASC specks formation in BMDMs deleted of Nrf2, primed with LPS, and followed by stimulation with Nigericin, ATP and MSU. Scale bar, 20 μm. **e** Immunoblot analysis of ASC oligomerization in cross-linked cytosolic pellets of BMDMs deleted of Nrf2, primed with LPS, and followed by stimulation with Nigericin, ATP and MSU. Data are from three independent experiments (**a, b, d**, Values are mean ± SD) or are representative of three independent experiments with similar results. Student' s *t* test (**a, b, d**), Two-way ANOVA (**e**), ***P <0.001, ns not significant.

Subsequently, we sought to investigate the underlying mechanism by which Nrf2 regulates NLRP3 inflammasome activation. Given that NLRP3 inflammasome assembly is an essential step in NLRP3 inflammasome full activation, we tested the effects of Nrf2 on this process. The NLRP3 inflammasome assembly is controlled by upstream signaling events, especially potassium efflux and chloride ion efflux [11, 12, 26]. We found that nigericin stimulation resulted in a significant increase in potassium and chloride ion efflux, and deleting Nrf2 showed only a tenuous effect on this tendency (Fig. 2a, b), suggesting that Nrf2 did not affect these upstream signaling events. During the activation stage, the adaptor protein ASC self-aggregates to form ASC specks and ASC oligomers, which are decisive for NLRP3 inflammasome assembly completion [27]. Accordingly, we wanted to determine the role of Nrf2 in ASC specks and oligomerization formation by a well-established biochemical assay [11, 12, 15, 16]. As expected, results showed that deleting Nrf2 dramatically inhibited the ASC specks formation induced by nigericin, ATP, or MSU in BMDMs (Fig. 2c, d). Additionally, ASC oligomerization formation induced by nigericin, ATP, or MSU was also inhibited in Nrf2^–/–^ BMDMs (Fig. 2e). These data indicate that Nrf2 is required for NLRP3 inflammasome assembly. Taken together, these results suggest that Nrf2 may promote NLRP3 inflammasome activation by facilitating NLRP3 inflammasome assembly.

### Klf9 is the target for Nrf2 during NLRP3 inflammasome activation

**Fig. 3:**
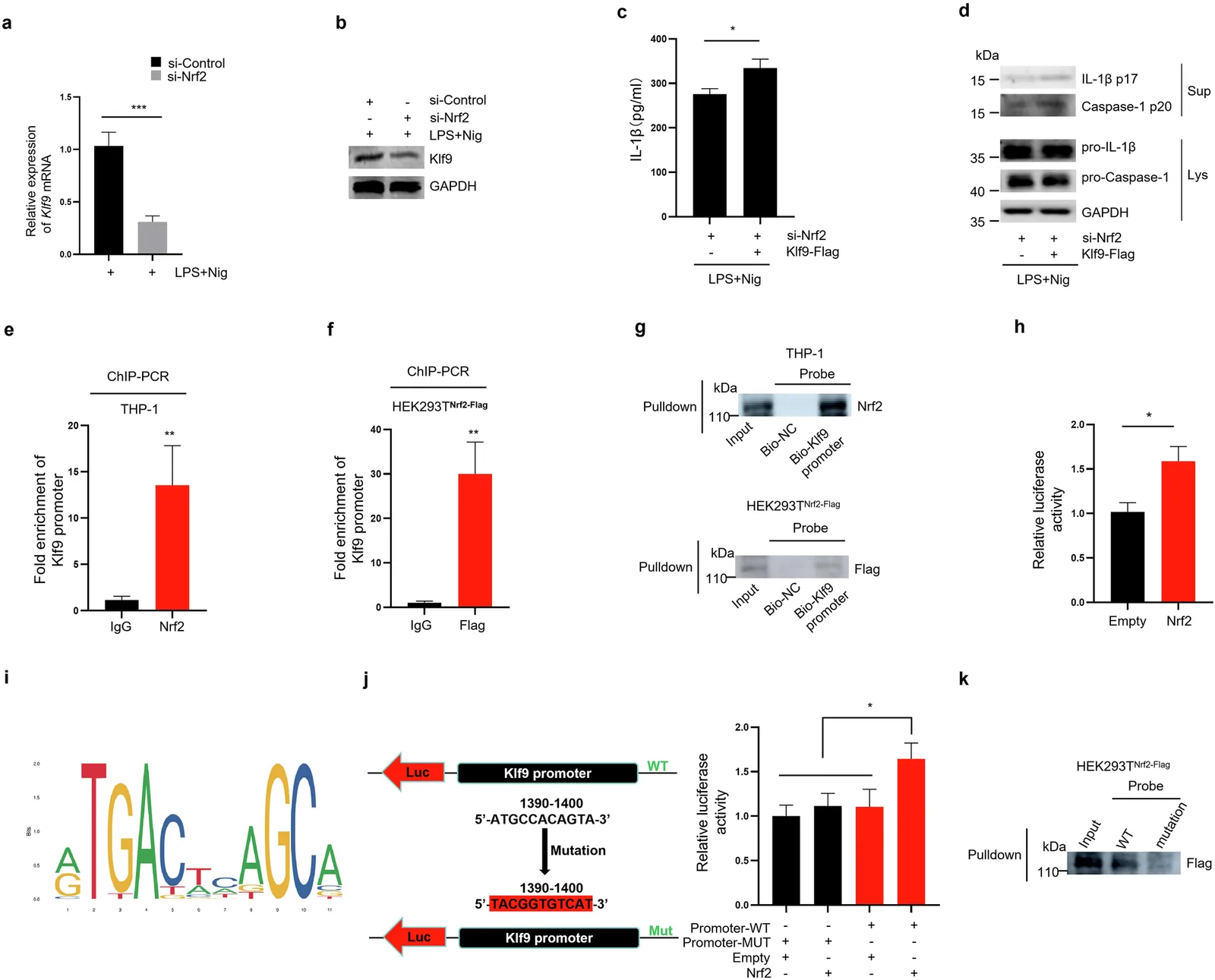
Klf9 is the target for Nrf2 during NLRP3 inflammasome activation. RT-PCR analysis (**a**) or immunoblot analysis (**b**) of Klf9 expression from THP-1 cells, primed with LPS, and followed by stimulation with Nigericin. **c** ELISA of IL-1β in supernatants from knockdown Nrf2 THP-1 cells transfected with Klf9-Flag, primed with LPS, and followed by stimulation with Nigericin. **d** Immunoblot analysis of supernatants or cell lysates from knockdown Nrf2 THP-1 cells transfected with Klf9-Flag, primed with LPS, and followed by stimulation with Nigericin. ChIP experiments were performed to detect the direct binding between Nrf2 and Klf9 promoter using Nrf2-specific antibody in THP-1 cells (**e**), or Flag-specific antibody in HEK293T cells transfected with Nrf2-Flag (**f**). **g** DNA pulldown was performed to detect the association of the Klf9 promoter with Nrf2 using a specific Klf9 promoter probe in THP-1 cells(upper), or a Flag-specific antibody in HEK293T cells transfected with Nrf2-Flag(below). **h** Luciferase activities were performed to detect the promoting effects of Nrf2 on Klf9 transcription. **i** The predicted Nrf2-binding motif. **j** Luciferase activities were performed using wild-type or mutant Klf9 promoter to confirm the binding sites of Klf9 promoter to Nrf2. **k** DNA pulldown was performed using a specific probe with wild-type or mutant Klf9 promoter in HEK293T cells transfected with Nrf2-Flag. Data are from three independent experiments (**a, c, e, f, h, j**, Values are mean ± SD) or are representative of three independent experiments with similar results. Student' s *t* test(**a, c, e, f, h, j**), Two-way ANOVA (**l**), *P < 0.05, ***P < 0.001, ns not significant.

Next, we attempted to analyze the specific mechanism by which Nrf2 facilitates NLRP3 inflammasome assembly. There is a common manner in which regulators directly interact with components of the NLRP3 inflammasome to affect its assembly and subsequent activation [27–30]. We hypothesized that Nrf2 interacts with components closely related to NLRP3 inflammasome activation, including NLRP3, ASC, pro-caspase-1, and NEK7. We expressed Flag-tagged Nrf2 and VSV-tagged NLRP3, His-tagged ASC, HA-tagged pro-caspase-1, and Myc-tagged NEK7 in HEK293T cells and performed co-immunoprecipitation (Co-IP) assays. Surprisingly, the Co-IP assay showed that Nrf2 did not interact with NLRP3, ASC, pro-caspase-1, or NEK7 (Supplementary Fig. 2a–d), suggesting that Nrf2 does not directly facilitate NLRP3 inflammasome assembly. Thus, we next searched for the target for Nrf2. Considering that Nrf2 is a transcriptional factor in multiple physiological responses [17, 18, 31, 32]. We adopted RNA-seq to profile Nrf2 target genes in THP-1 cells with or without Nrf2 knockdown. Differentially expressed genes (DEGs) were identified as those with fold changes > 2 and *P* < 0.05. Based on the RNA-seq results, 153 DEGs were upregulated and 94 DEGs were downregulated due to Nrf2 knockdown in the cell model of NLRP3 inflammasome activation (Supplementary Fig. 3a). The downregulated genes were further analyzed, and *Klf6, TNFSF18, Klf9*, and *TEX54* were chosen as the top-ranked genes that exhibited significant differences (Supplementary Fig. 3b). Among these, no significant differences were found in *Klf6, TNFSF18*, or *TEX54* mRNA between THP-1 cells with and without Nrf2 knockdown stimulated with LPS plus nigericin, except for *Klf9* mRNA (Fig. 3a; Supplementary Fig. 3c–e). Klf9 protein in THP-1 cells was also decreased by knockdown of Nrf2 (Fig. 3b). These results suggest that Klf9 is a downstream target of Nrf2.

Next, we tested whether Klf9 is a participant in regulating NLRP3 inflammasome activation under stimuli. There was a significant decrease in caspase-1 activation and IL-1β secretion after silencing Klf9 by siRNA in THP-1 cells stimulated with nigericin, ATP, or MSU (Supplementary Fig. 4a–c), suggesting that Klf9 enhances NLRP3 inflammasome activation. In addition, ASC oligomerization formation induced by nigericin was disturbed in Klf9 knockdown THP-1 cells (Supplementary Fig. 4d). These results demonstrate that Klf9 promotes NLRP3 inflammasome assembly and activation. To explore whether Nrf2 promoters NLRP3 inflammasome activation by raising Klf9, we transfected a Flag-Klf9-expressing plasmid into Nrf2 knockdown THP-1 cells to enhance the expression of Klf9. We discovered that overexpression of Klf9 reversed the Nrf2-knockdown-induced reduction of caspase-1 activation and IL-1β secretion (Fig. 3c, d), supporting the idea that Nrf2 regulates NLRP3 inflammasome activation by increasing Klf9. Thus, these data demonstrate that Klf9 is the downstream target of Nrf2 during NLRP3 inflammasome activation.

Nrf2 has been reported to be a transcriptional regulator of Klf9 [33]. The aforementioned findings show that Nrf2 can regulate Klf9 at both the transcriptional and protein levels [34, 35]. Klf9 was shown to be a target gene of Nrf2 at Harmonizome (https://maayanlab.cloud/Harmonizome). However, the regulatory role of Nrf2 on Klf9 transcription was not identified. A ChIP-PCR assay with an Nrf2 antibody was performed in THP-1 cells and showed that endogenous Nrf2 is recruited to the *Klf9* promoter (Fig. 3e). To confirm this result, exogenous Nrf2 protein was expressed in HEK293T cells and ChIP-PCR confirmed that Nrf2 could be recruited to the *Klf9* promoter (Fig. 3f). Next, we generated a DNA probe with the *Klf9* promoter to perform a DNA pulldown analysis in THP-1 and HEK293T cells. The probe pulled down Nrf2 protein in THP-1 cells and Flag in HEK293T cells transfected with Nrf2-Flag (Fig. 3g). We constructed a luciferase reporter carrying the *Klf9* promoter and found that overexpression of Nrf2 enhanced the luciferase activity (Fig. 3h). These results demonstrate that Nrf2 acted as a transcriptional regulator of *Klf9* to boost its mRNA expression. We then explored the specific binding site of Nrf2 on the *Klf9* promoter. The JASPAR (https://jaspar.genereg. net/) and NCBI databases were used to predict binding sites of Nrf2 in the *Klf9* promoter sequence. We selected three sites with high scores, namely site 1 (Fig. 3j), site 2 (Supplementary Fig. 5a), and site 3 (Supplementary Fig. 5b) and constructed luciferase reporters including wild-type and mutant variants of the predicted binding sites of the *Klf9* promoter. Nrf2 overexpression increased the reporter activity of the *Klf9* promoter, whereas mutation of binding site 1 attenuated the activity mediated by Nrf2, while mutation of sites 2 and 3 barely affected the activity (Fig. 3j; Supplementary Fig. 5a, b). DNA probes targeting the mutated site 1 pulled down Nrf2 protein less than wild-type site 1 (Fig. 3k), suggesting that the Nrf2 binding site on the *Klf*9 promoter is site 1 (ATGCCACAGTA), located at –1390 to –1400. Collectively, these findings support that Nrf2 targets Klf9 to promote NLRP3 inflammasome activation.

### Klf9 mediates NLRP3 inflammasome activation by TXNIP

**Fig. 4:**
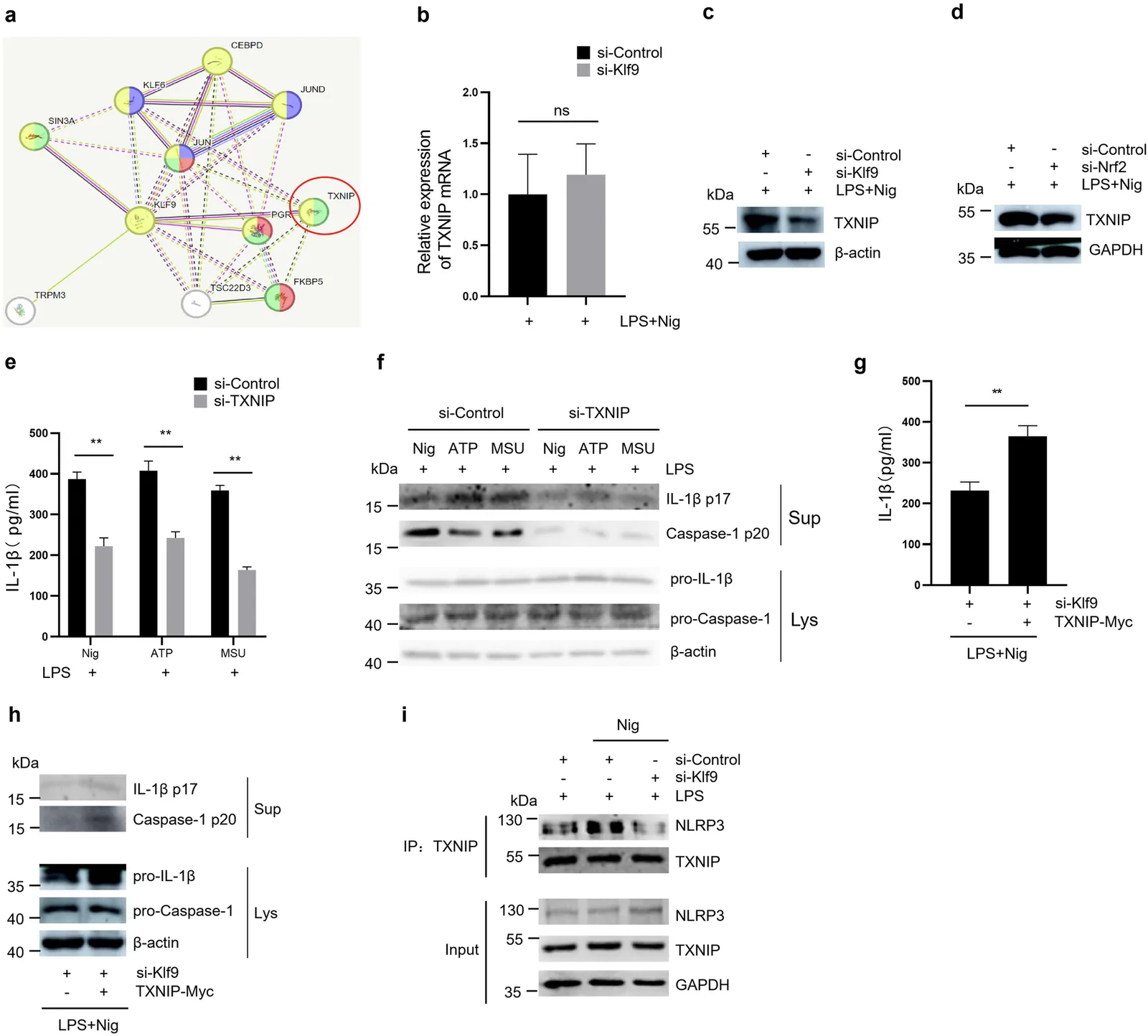
Klf9 mediates NLRP3 inflammasome activation by TXNIP. **a** String bioinformatics database (https://string-db.org/)analysis of the interaction protein of Klf9. **b** RT-PCR analysis of TXNIP from THP-1cells silenced of Klf9, primed with LPS, and followed by stimulation with Nigericin. **c** Immunoblot analysis of TXNIP in THP-1 cells silenced of Klf9, primed with LPS, and followed by stimulation with Nigericin. **d** Immunoblot analysis of TXNIP in THP-1 cells silenced of Nrf2. **e** ELISA of IL-1β in supernatants from THP-1 cells silenced of TXNIP, primed with LPS, and followed by stimulation with Nigericin, ATP and MSU. **f** Immunoblot analysis of supernatants or cell lysates from THP-1 cells silenced of TXNIP, primed with LPS, and followed by stimulation with Nigericin, ATP and MSU. **g** ELISA of IL-1β in supernatants from Klf9 knockdown THP-1 cells transfected with TXNIP-Myc, primed with LPS, and followed by stimulation with Nigericin. **h** Immunoblot analysis of supernatants or cell lysates from Klf9 knockdown THP-1 cells transfected with TXNIP-Myc, primed with LPS, and followed by stimulation with Nigericin. **i** IP and immunoblot analysis of the interaction of NLRP3 and TXNIP in THP-1 cells silenced of Klf9, primed with LPS, and followed by stimulation with Nigericin. Data are from three independent experiments (**b, e, g**, Values are mean ± SD) or are representative of three independent experiments with similar results. Student' s *t* test (**b, e, g**), Two-way ANOVA (**l**), **P < 0.01, ns not significant.

Although Klf9 has been reported to participate in NLRP3 inflammasome activation, Klf9 is not a direct regulator of NLRP3 inflammasome assembly [36]. We then determined how the Klf9 mediated NLRP3 inflammasome activation. By analyzing the String database (https://string-db.org/), we found that TXNIP is a vital facilitator of NLRP3 inflammasome activation [37]. may be interacting with Klf9 (Fig. 4a). Hence, we speculated that Klf9 may mediate NLRP3 inflammasome activation via TXNIP. We examined the effect of Klf9 on TXNIP expression and found that knockdown of Klf9 by siRNA decreased the TXNIP protein level but barely affected the mRNA level in NLRP3 inflammasome activation (Fig. 4b, c). Meanwhile, we found that the TXNIP expression is decreased under the Nrf2 knockdown (Fig. 4d), consistent with other studies [14, 37, 38]. depressing TXNIP significantly inhibited caspase-1 activation and the following IL-1β secretion in macrophages induced by stimuli of the NLRP3 inflammasome agonists (Supplementary Fig. 6; Fig. 4e, f). Next, a rescue test was implemented by transfecting the Myc-TXNIP plasmid into Klf9 knockdown THP-1 cells. Overexpression of TXNIP inhibited the Klf9-silencing-induced reduction in caspase-1 activation and the subsequent IL-1β secretion (Fig. 4g, h). Thus, these results indicate that TXNIP is required for Klf9 mediating NLRP3 inflammasome activation. As an NLRP3-binding protein, the interaction between TXNIP and NLRP3 is a prerequisite for NLRP3 inflammasome assembly and subsequent activation [39]. Our results showed that the TXNIP-NLRP3 interaction was increased during NLRP3 inflammasome activation; however, suppressing Klf9 significantly reversed the TXNIP-NLRP3 interaction (Fig. 4i). Accordingly, these findings suggest that Klf9 mediates NLRP3 inflammasome activation by sustaining the TXNIP-NLRP3 interaction.

### Klf9 stabilizes TXNIP via deubiquitination

**Fig. 5:**
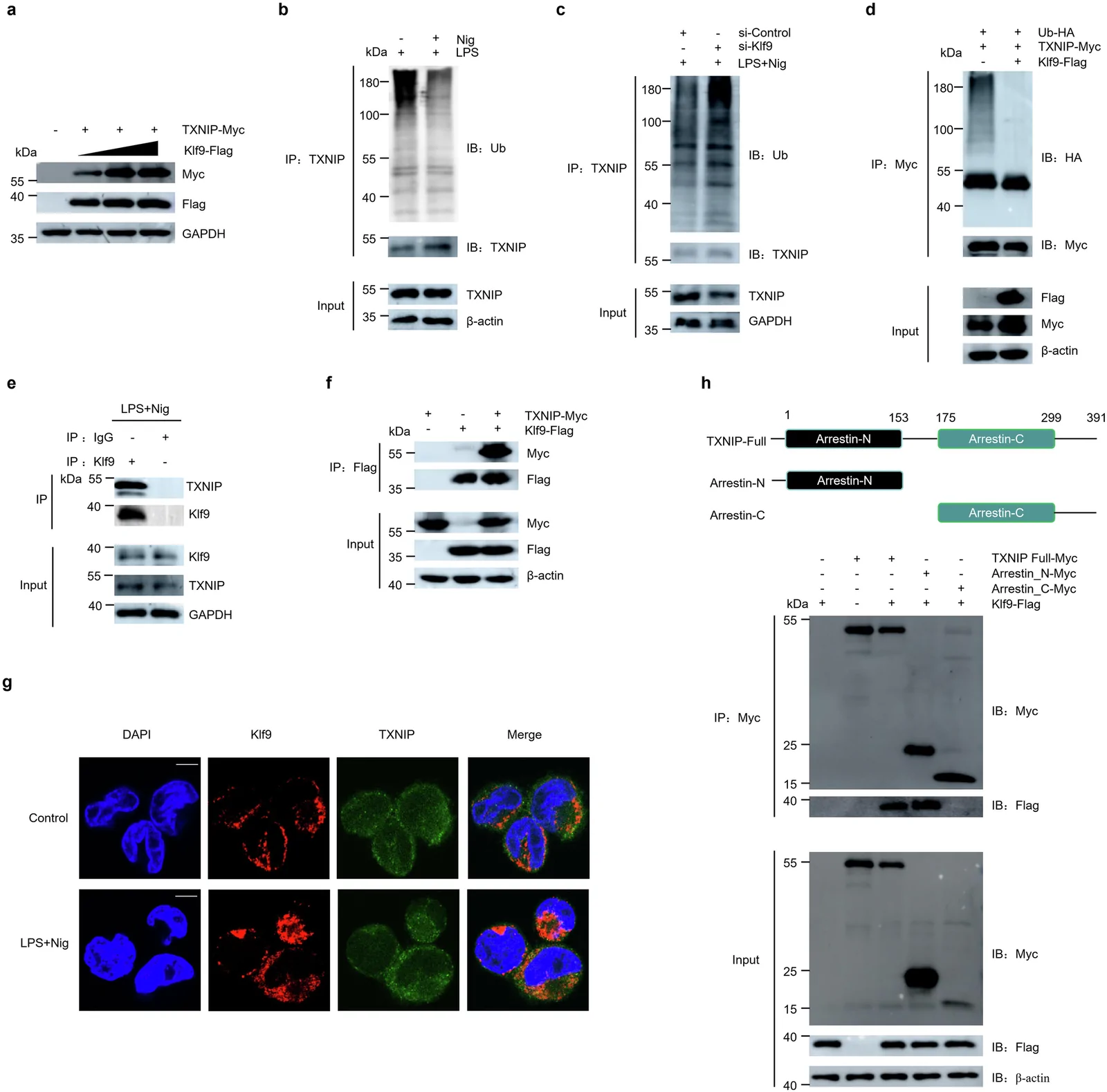
Klf9 stabilizes TXNIP via deubiquitination. **a** Immunoblot analysis of lysates from HEK293T cells transfected with TXNIP-Myc and increasing Klf9-Flag. **b** Immunoblot analysis of lysates from THP-1 cells treatment with LPS plus Nigericin, followed by IP with anti-TXNIP, probed with anti-ubiquitin(Ub). **c** Immunoblot analysis of lysates from THP-1 cells silenced of Klf9 treatment with LPS plus Nigericin, followed by IP with anti-TXNIP, probed with anti-Ub. **d** Immunoblot analysis of lysates from HEK293T cells transfected with TXNIP-Myc, Klf9-Flag and Ub-HA, followed by IP with anti-Myc, probed with anti-HA. **e** IP analysis of the interaction of Klf9 and TXNIP in THP-1 cells, primed with LPS, and followed by stimulation with Nigericin. **f** Immunoblot analysis of lysates from HEK293T cells transfected with TXNIP-Myc and Klf9-Flag, followed by IP with anti-Flag, probed with anti-Myc. **g** Representative immunofluorescence staining to test the colocalization of Klf9 and TXNIP in THP-1 cells, primed with LPS, and followed by stimulation with Nigericin. Scale bar, 10 μm. **h** Immunoblot analysis of lysates from HEK293T cells transfected with Klf9-Flag and TXNIP-Myc, or TXNIP-Arrestin N-Myc, or TXNIP-Arrestin C-Myc, followed by IP with anti-Myc, probed with anti-Flag. Data are representative of three independent experiments with similar results.

Since the knockdown of Klf9 decreased the protein level but not the mRNA level of TXNIP, we investigated whether Klf9 led to an accumulation of TXNIP. There was a significant increase in exogenous TXNIP protein expression with increasing amounts of Klf9 plasmid (Fig. 5a). Therefore, we considered that Klf9 led to TXNIP protein accumulation by increasing protein synthesis or decreasing protein degradation. We used cycloheximide (CHX), a protein synthesis inhibitor, to test whether Klf9 regulates TXNIP protein synthesis and found that the knockdown of Klf9 decreased the half-life of TXNIP (Supplementary Fig. 7a, b). In addition, knocking down Klf9 did not abate the protein levels of TXNIP under the condition that protein degradation was inhibited by the proteasome inhibitor MG132 (Supplementary Fig. 7c). These data demonstrate that Klf9 protects TXNIP from proteasomal degradation rather than elevating protein synthesis. Ubiquitination is a crucial process that disrupts protein stability and accelerates protein degradation, and deubiquitination is the opposite process [40]. The protein stability of TXNIP can be controlled by the ubiquitination or deubiquitination [40]. We supposed that Klf9 might modulate the deubiquitination of TXNIP. TXNIP ubiquitination was significantly inhibited in THP-1 cells under NLRP3 inflammasome activation; however, knockdown of Klf9 reversed this decrease (Fig. 5b, c). Meanwhile, we found that knockdown of Nrf2 also damaged the inhibition of TXNIP ubiquitination (Supplementary Fig. 7d). Exogenous TXNIP ubiquitination was analyzed by transfecting Ub, Klf9, and TXNIP plasmids into HEK293T cells. Consistently, TXNIP ubiquitination was inhibited upon Klf9 overexpression (Fig. 5d). These results suggest that Klf9 stabilizes TXNIP by suppressing its ubiquitination.

TXNIP was predicted to interact with Klf9 using the String database. We hypothesized that Klf9 may interact with TXNIP to directly regulate TXNIP protein stability. To identify this hypothesis, we performed a Co-IP assay using Klf9 and TXNIP antibodies. Co-IP assays displayed the interaction between Klf9 and TXNIP in THP-1 cells during NLRP3 inflammasome activation (Fig. 5e; Supplementary Fig. 7e). We expressed exogenous Klf9 and TXNIP proteins in HEK293T cells and identified the interaction between Klf9 and TXNIP (Fig. 5f; Supplementary Fig. 7f). Confocal microscopy also showed an increase in colocalization of Klf9 and TXNIP (Fig. 5g). To confirm which domain(s) of TXNIP was responsible for the interaction with Klf9, we used NCBI’s conserved domain prediction to construct two truncated mutants of TXNIP, containing the arrestin N (N terminal) and arrestin C (C terminal), respectively (Fig. 5h). The results indicated that the N terminal but not the C terminal was the specific binding domain responsible for the interaction with Klf9 (Fig. 5h). Collectively, these findings demonstrate Klf9 causes a deubiquitination of TXNIP to stabilize TXNIP, which promotes TXNIP-NLRP3 interaction.

### Klf9 recruits USP30 to deubiquitinate TXNIP

**Fig. 6:**
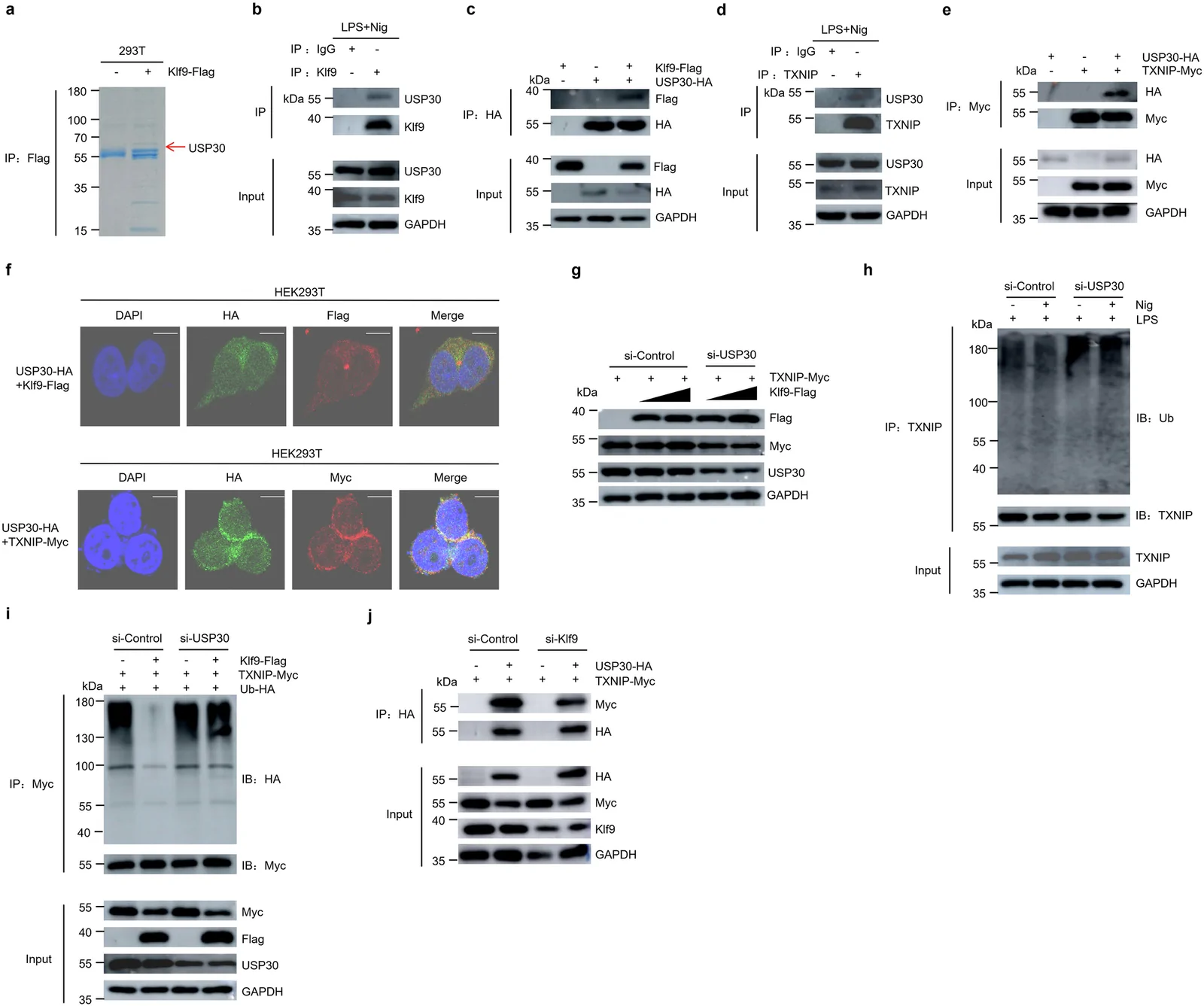
Klf9 recruits USP30 to deubiquitinate TXNIP. **a** The IP captured by anti-Flag from HEK293T cells transfected with Klf9-Flag was separated by SDS-PAGE gel and analysed by mass spectrometry. **b** Immunoblot analysis of lysates from THP-1 cells treated with LPS plus Nigericin, followed by IP with anti-Klf9, probed with anti-USP30. **c** Immunoblot analysis of lysates from HEK293T cells transfected with USP30-HA and Klf9-Flag, followed by IP with anti-HA, probed with anti-Flag. **d** Immunoblot analysis of lysates from THP-1 cells treated with LPS plus Nigericin, followed by IP with anti-TXNIP, probed with anti-USP30. **e** Immunoblot analysis of lysates from HEK293T cells transfected with USP30-HA and TXNIP-Myc, followed by IP with anti-Myc, probed with anti-HA. **f** Representative immunofluorescence staining showing the colocalization of exogenous USP30 and Klf9(upper), or USP30 and TXNIP(below) in HEK293T cells. Scale bar, 10μm. **g** Immunoblot analysis of lysates from USP30 knockdown HEK293T cells transfected with TXNIP-Myc and Klf9-Flag. **h** Immunoblot analysis of lysates from THP-1 cells silenced of USP30 treatment with LPS plus Nigericin, followed by IP with anti-TXNIP, probed with anti-Ub. **i** Immunoblot analysis of lysates from USP30 knockdown HEK293T cells transfected with TXNIP-Myc, Klf9-Flag and Ub-HA, followed by IP with anti-Myc, probed with anti-HA. **j** Immunoblot analysis of lysates from Klf9 knockdown HEK293T cells transfected with USP30-HA and TXNIP-Myc, followed by IP with anti-HA, probed with anti-Myc. Data are representative of three independent experiments with similar results.

No studies have reported that Klf9 functions as a deubiquitinase to stabilize target proteins. We investigated the mechanism by which Klf9 regulates TXNIP deubiquitination. We performed immunoprecipitation-mass spectrometry (IP-MS) analysis to detect proteins that potentially interact with Klf9 (Fig. 6a). Among the proteins screened, USP30 was chosen for further research because USP30 was the top-ranked deubiquitinase, and the molecular weight of USP30 matched the Coomassie staining stripe (Fig. 6a; Supplementary Fig. 8a, b). The interaction between Klf9 and USP30 was validated in THP-1 cells upon NLRP3 inflammasome activation (Fig. 6b). The interaction was confirmed via co-transfection of Klf9-Flag and TXNIP-Myc plasmids in HEK293T cells (Fig. 6c). Interestingly, an interaction between TXNIP and USP30 was observed (Fig. 6d, e). Exogenous colocalization between Klf9 and USP30, TXNIP and USP30 was confirmed by confocal microscopy, indicating that USP30 could interact with both Klf9 and TXNIP simultaneously (Fig. 6f).

Based on the above results, we speculated that Klf9 likely suppresses TXNIP ubiquitination in a USP30-dependent manner. To test this, we depleted USP30 in HEK293T cells and determined whether the TXNIP degradation controlled by Klf9 was affected. Compared with control cells, knocking down USP30 eliminated the Klf9-mediated inhibition of TXNIP protein degradation (Fig. 6g). Knockdown of USP30 significantly enhanced the ubiquitination of TXNIP in THP-1 cells upon NLRP3 inflammasome activation (Fig. 6h). We expressed Klf9, TXNIP, and ubiquitin in HEK293T cells with si-USP30 and found that decreasing USP30 damaged the deubiquitination of TXNIP induced by Klf9 (Fig. 6i). We tested whether the interaction between TXNIP and USP30 was regulated by Klf9 and found that knockdown of Klf9 suppressed the interaction between TXNIP and USP30 (Fig. 6j). Take together, these data demonstrate that Klf9 mediates TXNIP deubiquitination by recruiting USP30.

### Nrf2 deficiency ameliorates NLRP3 inflammasome activation in vivo

**Fig. 7:**
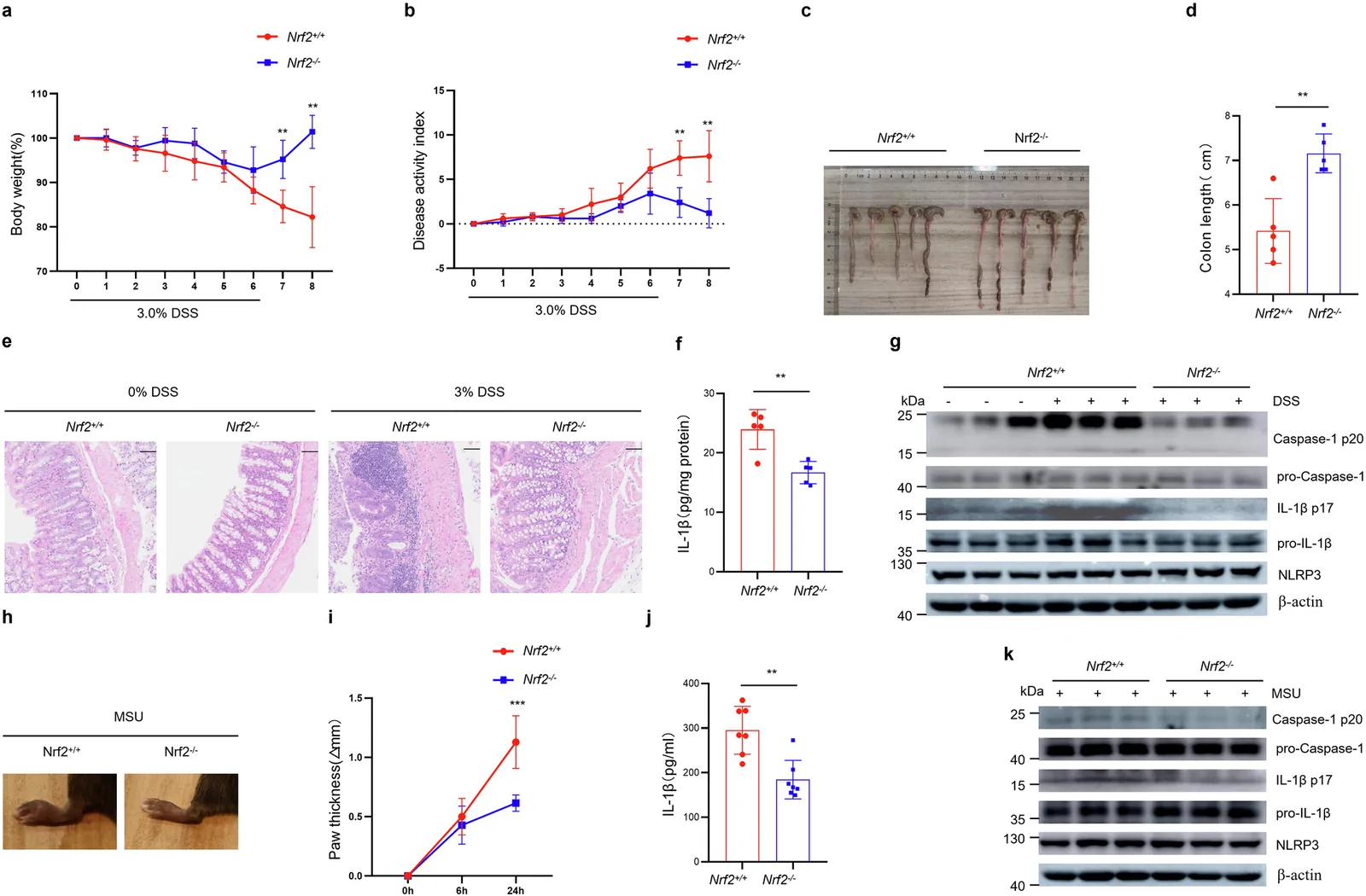
Nrf2 deficiency ameliorates NLRP3 inflammasome activation in vivo. Mice were administered with 3.0% DSS in drinking water for 6 days, followed by regular drinking water for 2 days(*n* = 5 mice). **a** Body weight. **b** Diseases activity. **c, d** Colon length. **e** Histopathological changes in colon tissue were examined by H&E staining. Scale bar, 100μm. **f** ELISA of IL-1β in cultured supernatants of colon tissues. **g** Immunoblot analysis of lysates from colon tissues. MSU crystals were injected into the footpads of mice for 24 h (*n* = 7 mice). **h** Representative macroscopic images of hind limbs. **i** Paw swelling. **j** ELISA of IL-1β in cultured supernatants of footpad tissues. **k** Immunoblot analysis of lysates from footpad tissues. Student' s *t* test (**a, b, d, f, i, j**), **P < 0.01.

Considering that Nrf2 promoted NLRP3 inflammasome activation in vitro, we tested whether depressing Nrf2 would inhibit NLRP3 inflammasome activation in vivo. First, DSS-induced colitis, widely used to study inflammatory bowel disease, was employed to assess the role of Nrf2 in intestinal inflammation [41]. Age- and sex-matched Nrf2^+/+^ and Nrf2^–/–^ mice were treated with drinking water containing 3% DSS to establish colitis. Notably, Nrf2^–/–^ mice exhibited significantly attenuated body weight loss compared with wild-type counterparts (Fig. 7a). Disease activity scores, reflecting clinical colitis severity, were also markedly reduced in Nrf2^–/–^ mice (Fig. 7b). Furthermore, colon shortening, a hallmark of severe colitis, was notably alleviated in Nrf2^–/–^ mice (Fig. 7c, d). Histopathological analyses confirmed that colonic epithelial integrity was substantially preserved in Nrf2^–/–^ mice, which characterized by reduced infiltration of inflammatory cells, fewer ulcers, and better maintenance of crypt structures compared to Nrf2^+/+^ mice (Fig. 7e). Additionally, both ELISA and Western blot analyses demonstrated that caspase-1 activation and IL-1β production were decreased in colonic tissues from Nrf2-deficient mice following DSS treatment (Fig. 7f, g). Collectively, these findings suggest that loss of Nrf2 provides protective effects against DSS-induced colitis.

To further validate the regulatory function of Nrf2 in vivo, an MSU crystal-induced acute gouty arthritis model, dependent on NLRP3 inflammasome activation, was also employed [8, 42]. Twenty-four hours post-injection of MSU crystals, paw swelling, a direct indicator of arthritis severity, was significantly reduced in Nrf2^–/–^ mice compared to Nrf2^+/+^ controls (Fig. 7h, i). Correspondingly, IL-1β secretion was notably lower in paw homogenates from Nrf2^–/–^ mice (Fig. 7j). Furthermore, western blot analysis indicated that caspase-1 activation and IL-1β production induced by MSU crystals were markedly attenuated in Nrf2^–/–^ mice (Fig. 7k). Together, these results confirm that Nrf2 promotes inflammatory responses through NLRP3 inflammasome activation in vivo, highlighting its potential as a therapeutic target in diseases driven by aberrant inflammasome activation, for example inflammatory bowel disease and gouty arthritis.

## Discussion

**Fig. 8:**
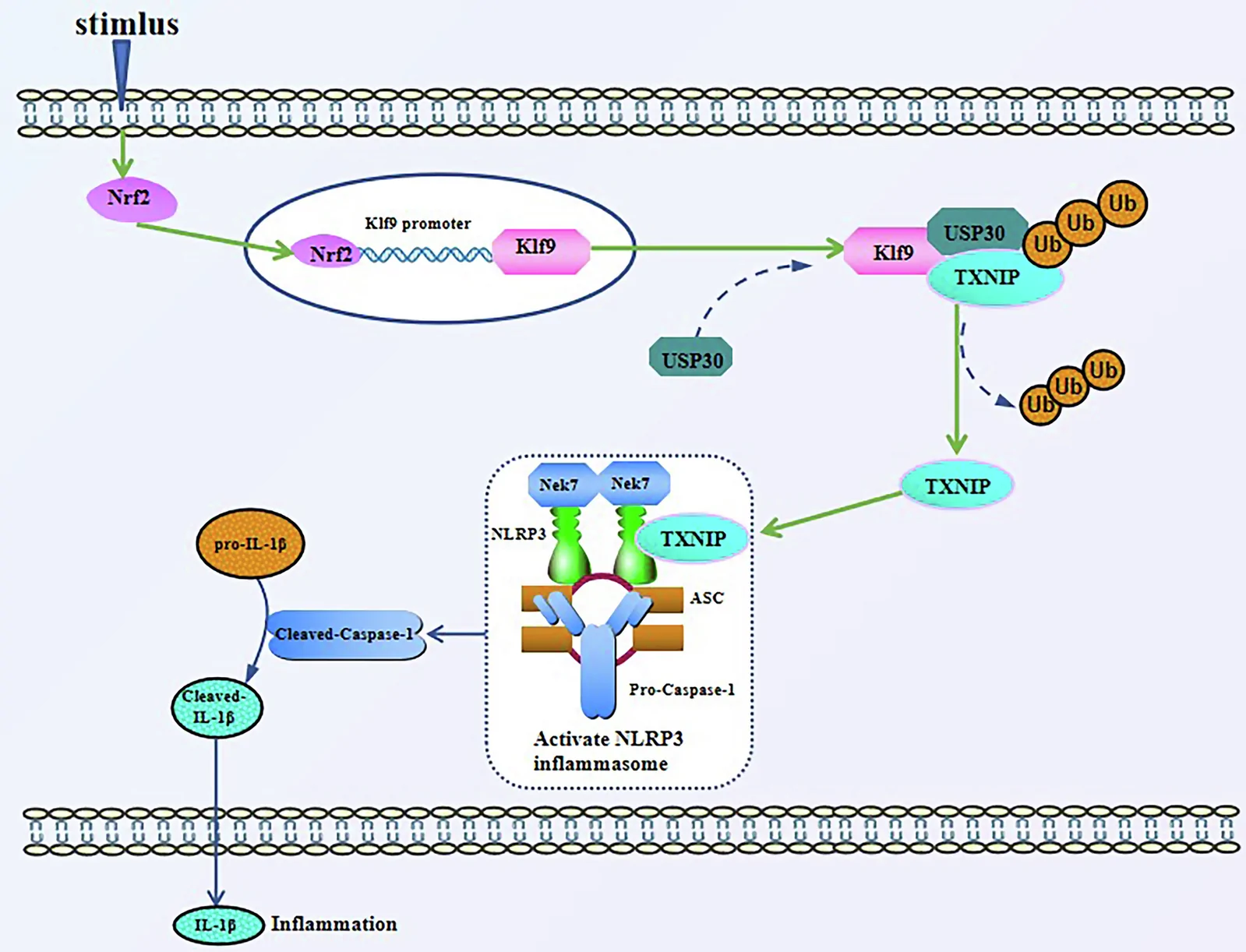
A model of NLRP3 inflammasome regulation by Nrf2. Nrf2 transfers to the nucleus and binds the promoter of Klf9 to raise Klf9 expression. Klf9 suppresses the protein degradation of TXNIP via a deubiquitinating pathway depending on recruiting USP30, which encourages the TXNIP-NLRP3 interaction. Subsequently, this response promotes the assemble and full activation of NLRP3 inflammasome.

The NLRP3 inflammasome is an indispensable component of innate immunity that supports host defense; however, its aberrant activation triggers multiple inflammatory diseases. Although intensively investigated over the past decades, the biological process of NLRP3 inflammasome activation remains unresolved. Although the best-known function of Nrf2 is its cellular antioxidant effects under various harmful stimuli, including inflammation, its function appears to be more diverse [18, 43–45]. Here, we show that Nrf2 is a facilitator of NLRP3 inflammasome activation via promoting the assembly step, which is in accordance with the view of previous research that Nrf2 is required for NLRP3 inflammasome activation [20–22, 46]. We also reveal a novel mechanism by which Nrf2 activates NLRP3 inflammasome. The results presented in this study established that Nrf2 raises Klf9 and Klf9 suppresses protein degradation of TXNIP via a deubiquitinating pathway. As an NLRP3-binding protein, TXNIP mediates NLRP3 inflammasome assembly by binding NLRP3 [38]. Based on these experimental data, we drew a model figure to illustrate how Nrf2 positively regulates NLRP3 inflammasome activation (Fig. 8).

The assembly process is indispensable and decisive for the full activation NLRP3 inflammasome and involves numerous potential therapeutic targets for NLRP3 inflammasome-related diseases [1, 2, 7, 8]. It should be noted that oxidative stress is the driving force of NLRP3 inflammasome activation by triggering its assembly [16]. Although Nrf2 is a well-known intracellular antioxidant [17]. The role of Nrf2 on NLRP3 inflammasome activation is controversial and remains unanswered. A previous study reported that Nrf2 could constrain NLRP3 inflammasome activation via inhibition of the priming signal [47]. However, other studies have confirmed that Nrf2 is required for NLRP3 inflammasome activation triggered by cholesterol crystals [20]. MSU [21]. ATP, or nigericin [22]. In our study, we identify that Nrf2 promotes NLRP3 inflammasome activation by facilitating NLRP3 inflammasome assembly. Consistent with previous research [20–22]. Our data support that Nrf2 is required for NLRP3 inflammasome activation. These findings also suggest that Nrf2 has extensive biological functions.

Although studies have explored the role of Nrf2 in NLRP3 inflammasome activation, how Nrf2 promotes NLRP3 inflammasome activation has not been thoroughly examined in these studies [20–22]. In the present study, we confirm that Nrf2-mediated upregulation of Klf9 mediates NLRP3 inflammasome activation using RNA-seq and other tests. We found that Nrf2 is recruited to the *Klf9* promoter and boosts Klf9 mRNA and protein expression. The Nrf2 binding site (ATGCCACAGTA) on the *Klf9* promoter is located at –1390 to –1400. Klf9 is an ubiquitously expressed member of an evolutionarily conserved transcriptional regulator KLF family [48]. Klf9 shows a key role in cell development, differentiation, growth and death by modulating gene transcription [49]. Nrf2 can regulate Klf9 expression, and Nrf2 interaction sites have been identified in the Klf9 promoter [34, 35]. Recently, a study reported that Nrf2 enhances the expression of Klf9 in response to severe oxidative stress [50]. We found that overexpression of Klf9 damaged the Nrf2-knockdown-induced reduction of caspase-1 activation and IL-1β secretion, which indicates that Klf9 is the downstream target of Nrf2 during NLRP3 inflammasome activation.

In general, Klf9 plays its role as a transcriptional regulatory factor [48, 49]. However, we identify a previously unknown function for Klf9 in this study. We discovered that Klf9 mediates NLRP3 inflammasome activation by stabilizing TXNIP, a vital activator of the NLRP3 inflammasome. TXNIP has a critical effect in activating the NLRP3 inflammasome; there are similarities in the phenotypes of caspase-1 activation and the following IL-1β secretion in NLRP3- and TXNIP- deficient mice [37]. As a binding partner of thioredoxin, TXNIP forms a complex with reduced thioredoxin, which prevents NLRP3 inflammasome activation under normal conditions [37, 51]. Interestingly, TXNIP is also a binding protein of NLRP3 [38]. Under detrimental stimulatory conditions, TXNIP dissociates from its complex with thioredoxin, binds to NLRP3 and then promotes NLRP3 inflammasome assembly and activation [37, 38]. It is beneficial for preventing NLRP3 inflammasome-driven inflammation, as it effectively inhibits the binding between TXNIP and NLRP3 [52]. Our data show that reducing Klf9 significantly impairs the TXNIP-NLRP3 interaction. These results suggest that Klf9 promotes the TXNIP-NLRP3 interaction during NLRP3 inflammasome activation. We also demonstrate that Klf9-induced stabilization of TXNIP may involve deubiquitinating TXNIP. Ubiquitination and deubiquitination are crucial post-transcriptional modifications of TXNIP [40, 53]. The deubiquitinase USP5 interacts with TXNIP and stabilizes it via deubiquitination in liver cells [40]. Considering that Klf9 has not been reported to function as a deubiquitinase that influences protein stability, we further explored how Klf9 regulates deubiquitination of TXNIP. We identified a deubiquitinase, USP30, that binds Klf9 and is recruited to TXNIP to inhibit TXNIP deubiquitination. These results indicate that USP30 is indispensable for Klf9-mediated deubiquitination of TXNIP. Collectively, our findings suggest that Klf9 stabilizes TXNIP via deubiquitination, which could promote TXNIP-NLRP3 interaction. We suppose that this is a pathway through which Klf9 mediates NLRP3 inflammasome activation.

Increasing evidence strongly indicates that blocking activation of the NLRP3 inflammasome is a promising therapeutic approach for treating related diseases [3, 5–7]. Some approaches have been invested with great effort, especially the development of small-molecule inhibitors to eliminate NLRP3 inflammasome activation [54–56]. However, most have failed in clinical trials. Previous studies have demonstrated that loss of Nrf2 attenuates atherosclerosis [20]. and alum-induced peritonitis [22]. In our study, data show that Nrf2- deficient mice show a protective effect against DSS-induced colitis and MSU crystals-induced acute gouty arthritis. Loss of Nrf2 also inhibits NLRP3 inflammasome activation in these mouse models. Hence, Nrf2 may be a therapeutic target for NLRP3 inflammasome-related diseases.

Several limitations of this study should be acknowledged. First, the functional impact of Klf9-TXNIP has been primarily assessed in cells. Future studies should investigate whether both Klf9 and TXNIP are targets for inhibiting NLRP3 inflammasome-mediated diseases in vivo. Additionally, the Nrf2 knockout is whole body but not macrophage-specific in vivo experiments. Considering that the investigations of Nrf2 regulating NLRP3 inflammasome activation are performed in macrophages, the results from mice with macrophage-specific knockout of Nrf2 may be more convincing. In summary, our findings again confirm the promoting role of Nrf2 in NLRP3 inflammasome activation. We reveal a previously unidentified regulatory mechanism for Nrf2 in NLRP3 inflammasome activation as a regulator of the Klf9-TXNIP axis. We demonstrate that Nrf2 raises Klf9 by binding to the *Klf*9 promoter, and Klf9 stabilizes TXNIP via deubiquitination, thereby promoting the TXNIP-NLRP3 interaction. The present study sheds valuable insights into developing therapeutic strategies targeting Nrf2 for NLRP3 inflammasome-mediated inflammatory diseases.

## Materials and methods

### Reagents and antibodies

Nigericin, MSU, phorbol-12-myristate-13-acetate (PMA), MG-132, and cycloheximide were from GlpBio (Montclair, USA). Adenosine triphosphate (ATP), disuccinimidyl suberate, anti-Flag, and anti-Myc were from Sigma (St. Louis, USA). Protein agarose, human IgG, penicillin/streptomycin, NP-40, protease and phosphatase inhibitors, and lipopolysaccharides (LPS) were from Beyotime Biotechnology (Shanghai, China). DAP1 was from Solarbio Science (Beijing, China). Anti-ASC(sc-514414), anti-NLRP3(sc-518122), anti-TXNIP(sc-271237), anti-Klf9(sc-376423), and anti-Nrf2(sc-365949) were from Santa Cruz Biotechnology (Dallas, USA). Anti-pro-Caspase-1 (WL02996), anti-pro-IL-1β(WL02257), and anti-IL-1β p17(WL00891) were from Wanleibio (Shenyang, China). Anti-Caspase-1 p20(AG-20B-0048-C100) was from Adipogen (San Diego, USA). Anti-GAPDH(60004), anti-β-actin(66009), horse anti-mouse IgG linked with horseradish peroxidase (HRP), and goat anti-rabbit IgG linked with HRP were from Proteintech (Wuhan, China). anti-HA(0906-1), anti-His(HA601079), FITC conjugated anti-mouse IgG(HA1128), and Alexa Fluor 647-conjugated anti-Rabbit IgG (H + L)(HA1123) were from Huabio (Hangzhou, China). Anti-GSDMD(39754) was from Cell Signaling Technology (Danvers, USA). Dextran sulfate sodium(DSS) was from AmBeed (Arlington, USA). Lipofectamine 3000 Transfection Reagent was purchased from Invitrogen (San Diego, USA).

### Mice

Wild-type (WT) C57BL/6 mice and Nrf2^–/–^ mice were purchased from Cyagen (Suzhou, China). Mice with Nrf2 deletion exhibited no clear physiological defects, and there were no obvious differences in body weight, size, and appearance between Nrf2 deletion and WT mice. Mice were maintained under standard pathogen-free conditions (humidity ~50%, temperature 24°C) with a regular 12-hour day/night cycle. All animal experiments and housing procedures were administered in strict conformity with protocols approved by the Institutional Animal Ethics Committee of Suining Central Hospital.

### Cell culture and treatments

HEK293T cells were cultured in DMEM containing 10% fetal bovine serum (FBS) and antibiotics (1% penicillin–streptomycin). Human THP-1 cells were cultured in RPMI 1640 medium supplemented similarly with 10% FBS and antibiotics (1% penicillin–streptomycin). THP-1 cells were differentiated with 100 nM PMA overnight to generate human macrophages. Primary bone marrow-derived cells (BMDMs) were obtained as previously described [57]. Briefly, BMDMs were isolated from the tibias and femurs of C57BL/6 J mice, and then the cells were cultured in DMEM containing 10% FBS, 1% penicillin–streptomycin and 30 ng/ml M-CSF for 5–6 days. THP-1 cells and BMDMs were cultured overnight, and cells were primed with LPS (300 ng/ml, 3 h), followed by treatment with nigericin (5 μM, 1 h), ATP (2.5 mM, 40 min), or MSU crystals (150 μg/ml, 24 h) to activate the inflammasome.

### Plasmids construction and transfection

The Nrf2-Flag plasmid was designed and synthesized from Fitgene Biotech (Guangzhou, China). NLRP3-VSV plasmid, ASC-His plasmid, pro-Caspase-1-HA plasmid, and NEK7-Myc plasmid were purchased from RealGene Biotech (Shanghai, China). Ub-HA plasmid, TXNIP-Myc plasmid, Klf9-Flag plasmid, USP30-HA plasmid, Arrestin N-Myc plasmid, and Arrestin C-Myc plasmid were purchased from Tsingke Biotech (Beijing, China). DNA sequencing was applied to test and confirm the constructs. HEK293T cells were transfected with plasmids using Lipofectamine 3000 following standard protocols.

### ELISA

Human IL-1β and IL-6 levels in cell culture supernatants were quantified using human ELISA kits from Multisciences(Hangzhou, China). Mouse IL-1β levels in culture supernatants were quantified using a mouse ELISA Kit (Neobioscience, Shenzhen, China). All tests were carried out according to the manufacturer’s instructions.

### Protein sample preparation and Western blot analysis

The total protein of the cell supernatant is prepared using the methanol/chloroform method as previously described [58, 59]. Cells were lysed in NP-40 lysis buffer with protease inhibitor and phosphatase inhibitor, and lysates were centrifuged at 10,000 × *g* at 4 °C for 10 min to obtain supernatants, and then the supernatants were used for Western blot analysis. The cytoplasmic protein and nuclear protein were isolated and collected by Nuclear and Cytoplasmic Protein Extraction Kit (Beyotime Biotechnology). Protein samples were separated by SDS–PAGE, followed by electrophoretic transfer onto PVDF membranes. Next, PVDF membranes were blocked with 5% nonfat milk and then incubated with primary antibodies at 4 °C overnight. Then, the blot was incubated with the appropriate secondary antibodies to detect protein levels.

### Immunoprecipitation

For co-immunoprecipitation assays, cells were lysed using NP-40 lysis buffer with protease and phosphatase inhibitors. The lysates of the cells were centrifuged at 10,000 × *g* at 4 °C for 10 min to prepare supernatants, and then the supernatants were incubated with Protein A + G Agarose and the specific antibody at 4 °C overnight. Next, the agarose was washed five times with PBS and eluted by boiling with SDS sample buffer at 100 °C for 10 min, and the samples were analyzed by immunoblotting.

### Immunofluorescence staining

THP-1 or HEK293T cells were seeded onto slides and cultured. Cells were fixed (4% paraformaldehyde, 20 min), permeabilized (0.3% Triton X-100, 10 min) and blocked (1% BSA, 1 h) at room temperature, followed by incubation with anti-Nrf2, anti-Flag or anti-Myc overnight at 4 °C. Then, cells were stained with the appropriate secondary antibodies linked with specific fluorescence and sealed with resin with DAPI. Stained cells were imaged under a confocal fluorescence microscope.

### Ubiquitination assay

Ubiquitination of TXNIP was measured using the following method. HEK293 cells were co-expressed Ub-HA, TXNIP-Myc, and Klf9-Flag, and treated with MG132 (5 μM, 4 h). Subsequently, cell lysates were collected, immunoprecipitated with an anti-Myc antibody, and subjected to immunoblot analysis with an anti-HA antibody. For endogenous TXNIP ubiquitination assays, THP-1 cells were processed similarly, then immunoprecipitated with an anti-TXNIP antibody and immunoblotted with an anti-Ub antibody.

### ASC oligomerization and ASC speck formation

THP-1 cells or BMDMs were treated and lysed in cold NP-40 lysis buffer for 30 min. The lysates were centrifuged at 330 × *g* for 10 min at 4 °C, and the pellets were collected and washed twice with ice-cold PBS and resuspended in PBS. The resuspended pellets were incubated with 2 mM disuccinimidyl suberate (DSS) at room temperature for 30 min with rotation to crosslink ASC. The samples were centrifuged at 1000 × *g* for 10 min, and the cross-linked pellets were obtained and re-suspended in 40 µl SDS sample buffer. Subsequently, samples were boiled at 100 °C for 10 min and analyzed via Western blotting. The ASC speck formation was analyzed by immunofluorescence staining as described above.

### LDH release assay

Pyroptosis was assessed by detecting lactate dehydrogenase (LDH) release into culture supernatants. Briefly, 120 μl of cell culture supernatant was moved to a new 96-well plate, followed by adding 60 μl LDH assay reagent (Beyotime Biotechnology) and co-incubation at 37 °C for 30 min. Absorbance at 490 nm was measured by a microplate reader. The release rate of cellular LDH was calculated according to the manufacturer’s instructions.

### RNA interference assay

SiRNAs were synthesized by Tsingke Biotech (Beijing, China) as follows: si-Nrf2: 5’-AAUACUUCUCGACUUACUC-3’, si-Klf9:5’-GUGCAGCGACAGUCUGGAA-3’, si-TXNIP: 5’-GAGAAUACAUGUUCCCGAA-3’, si-USP30: 5’-ACAGGAUGCUCACGAAUUA-3’. These siRNA duplexes were transfected into THP-1 or HEK293T cells using Lipofectamine 3000 reagent according to the manufacturer’s instructions.

### RNA quantitation

Total RNA was prepared using TRIzol Reagent (Thermo Fish, Waltham, US) following the manufacturer’s instructions. The quantitative RT-PCR analysis was performed with a LightCycler and a SYBR RT-PCR kit (TakaRa, Tokyo, Japan). All specific primers used for RT-PCR assays in this study were purchased from Sangon Biotech (Shanghai, China), as shown in Table S1.

### Chromatin immunoprecipitation

ChIP-PCR assays using a ChIP-kit (Beyotime Biotechnology) and PCR analysis were performed to test the interaction between Nrf2 and the Klf9 promoter. Anti-Nrf2, anti-Flag and control human IgG were applied.

### Measurement of intracellular chloride and potassium

Cell culture-grade water (200 µl) was added to THP-1 cells at 37 °C for 15 min to lyse the cells. The lysates were then centrifuged (10,000 × *g*, 5 min), and 50 µl supernatants were transferred to a new 1.5 ml EP tube, followed by incubation with incubated with 50 μl MQAE (10 μM). The fluorescence intensity was evaluated at excitation 350 nm and emission 460 nm by a microplate reader. The fluorescence intensity was evaluated at excitation 350 nm and emission 460 nm by a microplate reader. Intracellular potassium was measured using the EPG-2 AM potassium fluorescence indicator kit (Maokang Biotech, Shanghai, China). THP-1 cells were incubated with EPG-2 AM (5 μM) at 37 °C for 30 min. After removing the loading buffer, the probe’s fluorescence was visualized with a confocal microscope, and fluorescence intensity was quantified using ImageJ.

### Luciferase assay

Luciferase activities were evaluated through a Dual Luciferase Assay Kit (Promega, USA) following the manufacturer’s instructions. The luciferase reporter system was constructed by Tsingke Biotech (Beijing, China).

### Pulldown assay

Specific probes with wild-type or mutant *Klf9* promoters were constructed by Fitgene Biotech (Guangzhou, China). DNA pulldown assays were performed by incubating biotinylated DNA probes with cell lysates and streptavidin-coated magnetic beads. After washing the beads, bound proteins were eluted, separated by PAGE, and subsequently analyzed via Western blot.

### RNA-Seq and data processing

THP-1 cells with knocked-down Nrf2 or control were stimulated by LPS plus nigericin. Then, THP-1 cells were collected and resuspended in TRIzol reagent. Transcriptomic profiling was performed using RNA-seq analysis by Seqhealth Biotech (Wuhan, China). These data from RNA sequencing have been deposited in the Gene Expression Omnibus.

### Mass spectrometry analysis

HEK293T cells expressing Klf9-Flag were lysed using NP-40 lysis buffer containing protease inhibitor and phosphatase inhibitor, and then the supernatants were collected and incubated with Protein A + G agarose and anti-Flag antibody at 4 °C overnight. Next, the agarose was obtained and analyzed by immunoblotting. Protein samples were cut out of the gel (about 1 cm × 1 cm gel fragments) and analyzed by LC-MS. Mass spectrometry analysis was performed by Bioprofile Biotech (Shanghai, China).

### DSS-induced colitis

DSS was used to establish a colitis model. Firstly, 8-week-old WT or Nrf2-deficient C57BL/6 male mice were randomized and fed with drinking water containing 3.0% DSS for 6 days, and then fed with regular drinking water for 2 days. The mice were sacrificed on 8 day under anesthesia. The weight loss and overall disease severity of each mouse were assessed during this process, as previously described [41, 60]. Colon tissues of mice, being fixed in formalin and embedded in paraffin, were sectioned at 4 mm in thickness and subsequently stained with hematoxylin and eosin (H&E). The protein levels of NLRP3, caspase-1, and ASC in colon tissue were detected by immunoblot analysis.

### MSU-induced gout model

To establish a gout model induced by MSU, MSU crystals were resuspended in PBS (25 mg/ml), and 25 µl MSU crystal solution was randomized and injected into the plantar surface of the paws of WT or Nrf2-deficient C57BL/6 male mice. At the indicated time points, paw swelling was measured using an electronic caliper. Mice were sacrificed under anesthesia after injection of MSU crystals 24 h, and tissues were collected. The protein levels of NLRP3, caspase-1, and ASC in tissues were detected by immunoblot analysis.

### Statistical analysis

Data was expressed as the mean ± standard deviation. Statistical analysis was performed using GraphPad Prism 5.0. Statistical differences between groups were assessed using the two-tailed Student’s *t*-test or two-way ANOVA. A *P* value < 0.05 was considered to be statistically significant.

## Supplementary information

Supplementary Figure Legends

Supplementary Table 1

unprocessed images of Western blots

Supplementary Figure1

Supplementary Figure2

Supplementary Figure3

Supplementary Figure4

Supplementary Figure5

Supplementary Figure6

Supplementary Figure7

Supplementary Figure8

## Data Availability

The data that support the findings of this study are available from the corresponding author upon reasonable request.
