# Supplementary material for: Nrf2 promotes NLRP3 inflammasome assembly and activation by Klf9-TXNIP axis
Source: Cell Death Discov. 2026 Jun 26;12:365. doi: 10.1038/s41420-026-03219-3 (PMC13572388; doi:10.1038/s41420-026-03219-3)
Supplement: Supplementary file 1 [file 41420_2026_3219_MOESM1_ESM.doc]

**Supplementary Figure Legends**

**Supplementary Figure1.** **Immunoblot analysis of Nrf2 in THP-1 cells transfected with si-Nrf2**.

**Supplementary Figure2. The interaction between Nrf2 and components of NLRP3 inflammasome. (a-d)** Immunoblot analysis of lysates from HEK293T cells transfected with NLRP3-VSV and Nrf2-Flag, ASC-His and Nrf2-Flag, pro-Caspase-1-HA and Nrf2-Flag, or NEK7-Myc and Nrf2-Flag, followed by Immunoprecipitation(IP) with anti-Flag.

**Supplementary Figure3. The analysis of differentially expressed genes.** **(a-b)** Total RNAs were isolated from THP-1 cells with silencing Nrf2 treated with LPS plus Nigericin, and subjected to RNA-seq. Up-and down-regulated genes were shown by volcano plot(a). The differentially expressed genes shown by a heat map(b). **(c-e)** RT-PCR analysis of *Klf6*(d), *TNFSF18*(e), *TEX54*(f) from THP-1cells silenced of Nrf2, primed with LPS, followed by stimulation with Nigericin. Data are from three independent experiments (c, d, e, Values are mean±SD) . Student＇s *t*-test, *ns* not significant.

**Supplementary Figure4. Klf9 promotes NLRP3 inflammasome activation. (a)** Immunoblot analysis of Klf9 expression from THP-1 cells transfected with si-Klf9. **(b)** Immunoblot analysis of supernatants or cell lysates from THP-1 cells silenced of Klf9, primed with LPS, and followed by stimulation with Nigericin, ATP and MSU. **(c)** ELISA of IL-1β in supernatants from THP-1 cells silenced of Klf9, primed with LPS, and followed by stimulation with Nigericin, ATP and MSU. **(d)** Immunoblot analysis of ASC oligomerization in cross-linked cytosolic pellets of THP-1 cells silenced of Klf9, primed with LPS, and followed by stimulation with Nigericin. Data are from three independent experiments (c, Values are mean±SD) or are representative of three independent experiments with similar results. Student＇s *t*-test(c), Two-way ANOVA(l), ***P*＜0.01.

**Supplementary Figure5. Luciferase activities of different site of Klf9 promoter. (a-b)** Luciferase activities were performed using wild-type or mutant Klf9 promoter to confirm the binding sites of Klf9 promoter to Nrf2, respectively site2(a) and site3(b). Data are from three independent experiments (Values are mean±SD) . Student＇s *t*-test, *ns* not significant.

**Supplementary Figure6. Immunoblot analysis of TXNIP in THP-1 cells transfected with si-TXNIP**.

**Supplementary Figure7. Klf9 protects TXNIP from protein degradation via the ubiquitin proteasome pathway. (a-b)** Immunoblot analysis of half-life of TXNIP in THP-1cells silenced of Klf9 treatment with CHX(20μg/ml). **(c)** Immunoblot analysis of degradation of TXNIP protein from THP-1cells silenced of Klf9 treatment with CHX(20 μg/ml) and MG132 (5 μM). **(d)** Immunoblot analysis of lysates from THP-1 cells silenced of Nrf2 treatment with LPS plus Nigericin, followed by IP with anti-TXNIP, probed with anti-Ub. **(e)** Immunoblot analysis of lysates from THP-1 cells treatment with LPS plus Nigericin, followed by IP with anti-TXNIP, probed with anti-Klf9. **(f)** Immunoblot analysis of lysates from HEK293T cells transfected with TXNIP-Myc and Klf9-Flag, followed by IP with anti-Myc, probed with anti-Flag. Data are from three independent experiments (b, Values are mean±SD) or are representative of three independent experiments with similar results. Student＇s *t*-test(b), Two-way ANOVA(c), ***P*＜0.01.

**Supplementary Fig8. Mass spectrometry analysis. (a-b)** Analysis of mass spectrometry.
