# Supplementary material for: Nrf2 promotes NLRP3 inflammasome assembly and activation by Klf9-TXNIP axis
Source: Cell Death Discov. 2026 Jun 26;12:365. doi: 10.1038/s41420-026-03219-3 (PMC13572388; doi:10.1038/s41420-026-03219-3)
Supplement: Supplementary file 2 [file 41420_2026_3219_MOESM2_ESM.doc]

**Supplementary Table 1**

The sequences of this study.

| *TEX54* | Forward | GCCTTCTGATTACCGTGCTGTG |
| --- | --- | --- |
|  | Reverse | GAATCTGGTCTGGTTGCCTCTTG |
| *TNFSF18* | Forward | AGACTGCTAAGGAGCCCTGTATG |
|  | Reverse | CACGCAAGGAGGTTCAGAAGATG |
| *Klf6* | Forward | TCTGACAGCTCCGAGGAACTTTC |
|  | Reverse | GATGGAGGCGTGGAGGTGAC |
| *TXNIP* | Forward | TTCCTTACTGATCTATGTTAGCGTTCC |
|  | Reverse | GACCTGATCTGCTGCCAATTACC |
| *Klf9* | Forward | CTGGTTGCTGGGACTGTAGC |
|  | Reverse | GTTTTCCAGCTCCCAAACAG |
| *β-actin* | Forward | AGCCTCGCCTTTGCCGA |
|  | Reverse | CTGGTGCCTGGGGCG |
| *Klf9 promoter* | Forward | CGCTAGAGTTACGAAACAGGG |
|  | Reverse | GAAAGGCCATCCGTTCATGC |
| *GAPDH promoter* | Forward | TACTAGCGGTTTTACGGGCG |
|  | Reverse | TCGAACAGGAGCAGAGAGCGA |
